# Electrolyte disturbances and risk factors of acute kidney injury patients receiving dialysis in exertional heat stroke

**DOI:** 10.1186/s12882-016-0268-9

**Published:** 2016-06-06

**Authors:** Bancha Satirapoj, Suramanat Kongthaworn, Panbubpa Choovichian, Ouppatham Supasyndh

**Affiliations:** Division of Nephrology, Department of Medicine, Phramongkutklao Hospital and College of Medicine, 315, Bangkok, 10400 Thailand

**Keywords:** Exertional heat stroke, Acute kidney injury, Hypophosphatemia, Hypokalemia, Hyponatremia

## Abstract

**Background:**

Exertional heat stroke (EHS) is a life-threatening illness and leads to multi-organ dysfunction including acute kidney injury (AKI). The clinical significance of abnormal electrolytes and renal outcomes in ESH patients has been poorly documented. We aim to exhibit the electrolyte abnormalities, renal outcomes and risk factors of patients with AKI receiving dialysis in EHS.

**Methods:**

A retrospective cohort study in EHS patients between 2003 and 2014 were conducted. Clinical and laboratory outcomes including serum and urine electrolytes, AKI and dialysis were assessed on admission, during hospitalization and at the time of their discharge from the hospital. A logistic regression analysis was performed for risk factors of acute dialysis.

**Results:**

All 66 subjects with mean age 22.1 ± 4.3 years were included. On admission, the common electrolyte disturbances were hypokalemia (71.2 %), hypophosphatemia (59.1 %), hyponatremia (53.0 %), hypocalcemia (51.5 %), and hypomagnesemia (34.9 %). Electrolytes depletion was confirmed as renal loss (potassium loss; 54.2 %, phosphate loss; 86.7 %, sodium loss; 64.7 % and magnesium loss; 83.3 %). During hospitalization ranging from 2 to 209 days, 90.9 % patients suffered from AKI with 16.7 % receiving acute dialysis, and 3 % patients died. At discharge, AKI and electrolyte abnormalities had dramatically improved. The prognosis factors for AKI receiving dialysis were identified as neurological status, renal function and serum muscle enzyme at time of admission.

**Conclusion:**

The study suggests that hypoelectrolytemia and AKI are frequently observed in patients with EHS. Neurological impairment, impaired renal function, and increased serum muscle enzyme should be considered risk factors of acute dialysis.

## Background

Heat stroke is traditionally divided into exertional and classic types, and exertional heat stroke (EHS) is a recognized complication of intensive exercise training in humid and warm conditions. This life-threatening illness is encountered in tropical countries [1]. Despite aggressive treatment, heat stroke often produces high morbidity and mortality rates [2]. Clinical manifestations of EHS are related to the induction of a systemic inflammatory response such as early endogenous expression of interleukin-6 (IL-6) [3] and a disseminated intravascular coagulation triggered by heat stress, which may lead to multi-organ dysfunction including acute kidney injury (AKI) and electrolyte disturbances [4]. AKI following EHS is associated with increased mortality, and requires dialysis and longer hospital stays [5].

Heat related illness potentiates ischemic renal injuries, and is associated with temperature-induced oxidant stress during renal ischemia [6]. Abnormalities of electrolytes and renal function frequently develop in severe EHS patients [7, 8]. However, the effect of clinical and laboratory abnormalities on renal outcomes have been poorly addressed. Thus, we conducted a retrospective study from our hospital enrolling EHS patients admitted to the Department of Medicine. We assessed the electrolyte disturbances and incidence of AKI, examined variables associated with acute renal support and assessed their respective prognostic values during EHS.

## Methods

### Study design and participants

This research was a retrospective cohort study of a single center study of EHS patients admitted to the Department of Medicine, Phramongkutklao Hospital and College of Medicine, Bangkok, Thailand between January 2003 and January 2014. Criteria for diagnosis of heat stroke included a core temperature greater than 40 °C and central nervous system abnormalities presenting as delirium and coma with or without convulsions. All patients were evaluated with rectal temperature. Our treatment protocol included evaporative body heat loss by removing all of the patient's clothes and intermittently spraying the patient's body with warm water while a powerful fan blows across the body, gastric lavage with ice water, cold intravenous fluids, cooling blankets, and wet towels.

A detailed medical history was collected by reviewing medical records of the hospitals of all patients. Patient outcomes were followed up until discharge from the hospital. For the purposes of the present study, we excluded eight participants with missing serum electrolytes and creatinine information. All biochemical analyses of blood samples were conducted at the Phramongkutklao Hospital Laboratory. Serum creatinine was analyzed using the enzymatic method, calibrated to be traceable to isotope dilution mass spectrometry.

### Definition of electrolyte abnormalities, renal electrolytes loss and AKI

The reference range of serum sodium, potassium, calcium, phosphorus, and magnesium were 135 to 145 mEq/L, 3.5 to 5.5 mEq/L, 8.5 to 10.5 mg/dL, 2.5 to 5.5 mg/dL and 1.8 to 2.5 mg/dL, respectively. Values less than the lower range were considered as hypoelectrolytemia; and values greater than the upper range, as hyperelectrolytemia. The diagnosis of renal sodium, potassium, phosphate and magnesium loss were made in the presence of fraction excretion of sodium (FENa) >1 %, spot urine potassium (urine K) >15 mEq/L, fraction excretion of phosphate (FEPO_4_) >5 %, and fraction excretion of magnesium (FEMg) >2 %, respectively. AKI was defined as an abrupt increase in serum creatinine >0.3 mg/dL within 48 h, or serum creatinine increasing >1.5 times that of baseline which is known or presumed to have occurred within the prior 7 days.

### Statistical analysis

Data are expressed as mean ± SD, median and its 25 to 75 interquartile for nonGaussian variables (Kolmogorov-Smirnov test), or number and percentage. Statistical differences in variables were compared using independent Student’s *t*-test for normally distributed variables and the Kruskal-Wallis Test for non-normally distributed variables. We also performed a multiple forward logistic regression to assess variables associated with acute dialysis and calculated their hazard ratio and 95 % confidence interval (CI). All statistical tests were 2-sided, and *P* < 0.05 was required to reject the null hypothesis. Statistical analysis was performed using SPSS for Windows, version 12.0 (SPSS, Chicago, IL, USA).

## Results

The 66 subjects with EHS were screened for serum electrolytes and renal function on admission. The participants were all male Thais with mean age 22.1 ± 4.4 years. Body temperature was 40.8 ± 2.7 °C, Glasgow coma scale was 7.7 ± 3.5 and median serum creatinine was 1.6 mg/dL (interquartile range 0.9–12.7 mg/dL). Table 1 shows the clinical and laboratory characteristics at the time of admission.

**Table 1:** Baseline characteristics on admission

Initial clinical parameters	(*N* = 66)
Age (yr)	22.1 ± 4.4
Male (%)	100
Underlying disease: G6PD deficiency (%)	1.5
Body temperature (°C)	40.8 ± 2.7
Glasgow coma scale	7.7 ± 3.5
Median time to achieving BT of <38 °C (hr)	3.6 (1.8–8.5)
Pulse rate (/min)	132.5 ± 14.7
Mean arterial pressure (mmHg)	69.4 ± 8.7
Respiratory rate (/min)	26.2 ± 3.7
Blood tests	
Blood urea nitrogen (mg/dL)	14.1 (4.7–103)
Creatinine (mg/dL)	1.6 (0.9–12.7)
Sodium (mEq/L)	135.2 ± 5.8
Potassium (mEq/L)	3.4 ± 1.0
Chloride (mEq/L)	100.6 ± 7.8
Bicarbonate (mEq/L)	17.25 ± 3.93
Calcium (mg/dL)	8.3 ± 0.9
Phosphorus (mg/dL)	1.8 (0.4–14)
Magnesium (mg/dL)	2.0 ± 0.6
CPK (μg/L)	3671 (162–510680)
Glucose (mg/dL)	98.4 ± 9.3
Aspartate aminotransferase (U/L)	142.4 ± 61.5
Alanine aminotransferase (U/L)	96.1 ± 41.5
Alkaline phosphatase (U/L)	88.2 ± 12.6
Albumin (g/dL)	3.8 ± 0.3
Total bilirubin (μmol/L)	1.2 ± 0.4
Positive DIC profile (%)	42.4 %
Urine tests	
Urine pH	5.81 ± 0.78
Urine specific gravity	1.020 ± 0.01
Urine albumin (range 0–4)	1.0 (0–3)
Urine WBC >5/High power field (%)	45.4
Urine RBC >5/High power field (%)	72.7
Urine sodium (mEq/L)	109 ± 58.95
Urine potassium (mEq/L)	21.63 ± 21.22
Urine phosphate (mg/dL)	11.08 ± 9.84
Urine magnesium (mg/dL)	4.76 ± 6.36

Data are expressed as mean ± SD, median (25–75 interquartile), or number (%)

*CPK* Creatine phosphokinase, *G6PD* Glucose-6-Phosphate Dehydrogenase

On admission, the common electrolyte disturbances were hypokalemia (71.2 %), hypophosphatemia (59.1 %), hyponatremia (53.0 %), hypocalcemia (51.5 %) and hypomagnesemia (34.9 %) as shown in Table 2. Most of the electrolytes depletions were defined as renal tubular loss: renal potassium loss in 19 patients (54.2 %), renal phosphate loss in 13 patients (86.7 %), renal sodium loss in 11 patients (64.7 %) and renal magnesium loss in five patients (83.3 %).

**Table 2:** Electrolyte abnormalities on admission

	N/Total (%)
Hypernatremia	3/66 (4.5)
Hyponatremia	35/66 (53.0)
-Renal sodium loss (FENa > 1 %)	11/17 (64.7)
Hyperkalemia	3/66 (4.5)
Hypokalemia	47/66 (71.2)
-Renal potassium loss (Urine K > 15 mEq/L)	19/35 (54.2)
Hypercalcemia	-
Hypocalcemia	34/66 (51.5)
Hyperphosphatemia	5/66 (7.6)
Hypophosphatemia	39/66 (59.1)
-Renal phosphate loss (FEPO_4_ > 5 %)	13/15 (86.7)
Hypermagnesemia	6/66 (9.1)
Hypomagnesemia	23/66 (34.9)
-Renal magnesium loss (FEMg > 2 %)	5/6 (83.3)

Data are expressed as number (%)

During hospitalization with median time of 7 days, ranging from 2 to 209 days, 60 of 66 (90.9 %) patients presented AKI. AKI requiring dialysis developed in 11 of 66 (16.7 %) patients, and recovered to baseline in 63 of 66 cases (95.5 %). The mortality rate of the 66 patients was 3 %. At discharge status, median serum creatinine was 0.8 mg/dL (interquartile range 0.09–11.0 mg/dL) and EHS patients with AKI and electrolyte abnormalities dramatically improved with a range of these abnormalities between 1.5 and 22.7 %. Common electrolyte disturbances still persisted with hypocalcemia (22.7 %), hyponatremia (15.2 %) and hyperphosphatemia (7.6 %) (Table 3).

**Table 3:** Electrolyte abnormalities at discharges status and patient outcomes during hospitalization

	N/Total (%)
At discharges status	
Blood urea nitrogen (mg/dL)	9.9 (2.6–50)
Creatinine (mg/dL)	0.8 (0.09–11.0)
Hypernatremia	2/66 (3.0)
Hyponatremia	10/66 (15.2)
Hyperkalemia	-
Hypokalemia	4/66 (6.1)
Hypercalcemia	-
Hypocalcemia	15/66 (22.7)
Hyperphosphatemia	5/66 (7.6)
Hypophosphatemia	9/66 (13.6)
Hypermagnesemia	2/66 (3.0)
Hypomagnesemia	1/66 (1.5)
Renal and patient outcomes	
Acute kidney injury	60/66 (90.1 %)
Acute dialysis	11/66 (6.7 %)
Median length of stay in hospital (day)	7 (2–209)
Recovered acute kidney injury	63/66 (95.5 %)
Mortality rate	2/66 (3 %)

Data are expressed as median (25–75 interquartile), or number (%)

The comparison of EHS patients with and without acute dialysis is shown in Table 4. Neurological function (Glasgow coma scale), and serum bicarbonate on admission occurred significantly less among EHS patients with acute dialysis. In contrast, patients with acute dialysis presented significantly increased serum creatinine, phosphorus and creatine phosphokinase (CPK). The calculated risk factors for patients with AKI receiving dialysis were identified as rising serum creatinine (RR = 16.39, 95 % CI; 1.72–156.64, *P* = 0.015), hypernatremia (RR = 1.13, 95 % CI; 1.01–1.27, *P* = 0.041), hyperphosphatemia (RR = 1.66, 95 % CI; 1.19–2.33, *P* = 0.003) and hypermagnesemia (RR = 7.01, 95 % CI; 1.53–32.15, *P* = 0.012) and CPK > median value (RR = 13.91, 95 % CI; 1.66–116.41, *P* = 0.015). In contrast, high neurological function (Glasgow coma scale, relative risk (RR = 0.7, 95 % CI; 0.54–0.90, *P* = 0.007) and increased serum bicarbonate (RR = 0.68, 95 % CI; 0.53–0.86, *P* = 0.001) decreased risk factors for acute dialysis. By multivariate analysis, the significant predictors for acute dialysis were baseline neurological function, renal function and serum CPK level. (Table 5).

**Table 4:** Comparison of EHS patients with and without acute dialysis

	Non-dialysis (*N* = 55)	Acute dialysis (*N* = 11)	*P*-value
Age (yr)	22.2 ± 4.5	21.7 ± 3.7	0.775
Body temperature (°C)	40.8 ± 3.0	41.1 ± 0.7	0.738
Time to achieving Body temperature of <38 °C (hr)	3.3 (1.6–7.5)	3.8 (1.9–7.7)	0.572
Glasgow coma scale	8.3 ± 3.4	4.9 ± 2.7	0.003
Creatinine (mg/dL)	1.6 ± 0.4	5.3 ± 3.8	0.010
Sodium (mEq/L)	134.5 ± 4.9	138.6 ± 8.6	0.150
Potassium (mEq/L)	3.3 ± 0.6	4.0 ± 2.1	0.269
Bicarbonate (mEq/L)	18.1 ± 3.3	12.9 ± 4.3	0.001
Calcium (mg/dL)	8.4 ± 0.9	8.0 ± 1.5	0.432
Phosphorus (mg/dL)	1.9 ± 1.2	5.4 ± 4.6	0.035
Magnesium (mg/dL)	1.9 ± 0.3	2.5 ± 1.1	0.082
CPK (μg/L)	3123 (1898–8281)	31,688 (12,343–52,941)	<0.001

Data are expressed as mean ± SD, median (25–75 interquartile), or number (%). *P*-value corresponds to Independent t-test test and Kruskal-Wallis Test, *CPK* Creatine phosphokinase

**Table 5:** Multivariate logistic regression analysis predicting dialysis (*n* =66)

Variables	Unadjusted hazard ratio (95 % CI)	*p*-value	Adjusted hazard ratio (95 % CI)	*p*-value
Age (yr)	0.98 (0.83, 1.15)	0.771	-	
Body temperature (°C)	1.06 (0.73, 1.56)	0.746	-	
Glasgow coma scale	0.70 (0.54, 0.90)	0.007	0.67 (0.45, 0.99)	0.045
Creatinine (mg/dL)	16.39 (1.72, 156.64)	0.015	11.46 (1.05, 125.5)	0.046
Sodium (mEq/L)	1.13 (1.01, 1.27)	0.041	0.90 (0.68, 1.20)	0.478
Potassium (mEq/L)	1.80 (0.99, 3.25)	0.053	-	
Bicarbonate (mEq/L)	0.68 (0.53, 0.86)	0.001	0.75 (0.55, 1.01)	0.061
Calcium (mg/dL)	0.70 (0.37, 1.30)	0.255	-	
Phosphorus (mg/dL)	1.66 (1.19, 2.33)	0.003	1.22 (0.32, 4.61)	0.766
Magnesium (mg/dL)	7.01 (1.53, 32.15)	0.012	4.43 (0.18, 108.68)	0.362
CPK > median value (3577 μg/L)	13.91 (1.66, 116.41)	0.015	10.06 (1.15, 88.1)	0.037

Adjusted for significant factor in univariate analysis include Glasgow coma scale, serum creatinine, sodium, bicarbonate, phosphorus, magnesium and CPK (Creatine phosphokinase)

## Discussion

Based on our cohort study, hypoelectrolytemia with concurrent renal loss and kidney injury were frequently observed in patients with EHS at time of admission. These abnormalities dramatically improved after standard treatment. Baseline renal function, serum CPK, serum electrolytes disturbances including serum sodium, phosphate, magnesium, and bicarbonate and neurological status were a significant risk factor of acute dialysis in EHS patients.

Several studies have shown different electrolyte abnormalities during EHS [7–9]. Hyperthermia, hyperphosphatemia, hypocalcemia, hyperkalemia and acidosis are important abnormalities after events in patients with extensive skeletal muscle injury [10]. Our results were not consistent with those findings. Hypophosphatemia, hyponatremia, hypocalcemia, and hypokalemia were common at the time of admission of patients with EHS. Similarly, Tucker LE, et al. reported that in classical heat stroke hyponatremia, hypokalemia, hypocalcemia, hypomagnesemia, and hypophosphatemia were frequent but did not correlate with outcome [11]. In the series by Nalabawi et al., it was also apparent that hypophosphatemia (98 %), hypocalcemia (70 %) and hyponatremia (34 %), hypokalemia (32 %) and hypomagnesemia (30 %) were the common electrolyte abnormalities seen in classic heat stroke [7]. Finally, while reported in several series of exertional heat illness during military training, biochemical features have also been reported including dehydration and potassium depletion [12, 13].

Hypoelectrolytemia with tubular dysfunction and kidney injury developed in acute critical illness of EHS. Based on this phenomenon, it has been postulated that acute tubular injury comprises acute physiological changes associated with hyperthermia, the direct cytotoxicity of heat, the inflammatory and coagulation responses of the host resulting in injury to the vascular endothelium and kidney tissues [14, 15]. Hyperthermia precipitates ischemic AKI by magnifying the consequences of renal energy depletion with tubular membrane damage [16]. Evidence shows that pro-inflammatory cytokines released by leukocytes and renal tubular cells in severe heat illness could be important components of both the initiation and extension of kidney injury and dysfunction [17].

The direct stimulating effect of hyperthermia and acute critical illness on the respiratory center can be attributed to hyperventilation and transient hypophosphatemia [18]. Hypophosphatemia was observed within the initial phase of severe heat stroke, but hypocalcemia usually occurs on a later day [19]. Presumably, hypocalcemia resulted from precipitated in skeletal muscle injury. In our series, however, renal phosphate loss was the major mechanism of hypophosphatemia in the EHS setting. These results could be explained with the initial described mechanism of renal tubular injury.

Hypokalemia is also a common finding in EHS. It has previously been described that potassium depletion results in heat induced potassium shifting from hyperventilation, heat related sweating with hypovolemia and heat related renal injury. In an experimental study, high urine potassium excretion was demonstrated in the initial setting of renal ischemia in hypokalemic animals [20]. Our study reported that AKI was frequently observed in most patients with EHS at the time of admission. In addition, hypokalemia was accompanied by high urinary magnesium excretion. Based on this finding it has been hypothesized that potassium depletion could decrease reabsorption of magnesium through the paracellular pathway at the thick ascending limb of Henle’s loop, and thereby explaining the hypomagnesemia [21].

Renal injury is a well-documented feature in EHS. Our study demonstrated that AKI occurred frequently (90.1 %), AKI requiring dialysis had a significant incidence (16.7 %) and the majority of cases usually recovered full renal function (95.5 %). Similarly, long-term survival among patients with AKI due to rhabdomyolysis was close to 80 %, and most cases have good renal outcomes [22]. The etiology is multifactorial, and the main causes include direct thermal injury, rhabdomyolysis, renal hypoperfusion due to volume depletion and arterial hypotension [23] with increasing circulating vasopressor hormones and decreasing vasodilator hormones [24]. The hormonal changes can contribute to the pathophysiology of AKI in EHS. The clinical significance of serum electrolyte abnormalities during EHS has also been poorly documented. Previous study showed that elevated serum muscle enzymes, and electrolyte imbalances predicted the degree of renal injury and renal outcomes in patients with rhadomyolysis [25]. Our results confirmed that serum creatinine, sodium, phosphate, magnesium, bicarbonate, CPK and neurological status were associated with acute dialysis in EHS patients. After adjusting various confounders; however, only baseline renal function, serum levels of muscle enzyme and neurological status served as important factors in predicting acute dialysis in patients with AKI with EHS. These could explain that the degree of muscle injury related to elevated serum creatinine and muscle enzyme in EHS further increased the rate of patients with AKI on acute dialysis. Interestingly, one study confirmed the finding that the degree of muscle-damaging exercise before training in a heat environment results in increased systemic inflammatory response and a biomarker of kidney injury and excised induced muscle damage was one risk factor for developing AKI [26]. AKI is an essential feature in the EHS patients, suggesting that rhabdomyolysis played a central role in the pathophysiology of this complication.

Our study had some limitations. First, the multivariate analysis identifies a relationship between clinical/biochemical status and acute dialysis but does not infer causality. Second, some variables might have been missed especially urine electrolytes because of the retrospective study design. Third, we did not measure long term renal outcomes after discharge from hospital. Lastly, the exact mechanisms of renal electrolyte loss could not be determined because only spot urine samples in the oliguric EHS subjects were analyzed and novel urinary biomarker and hormonal analysis were lacking.

## Conclusion

Serum electrolyte abnormalities including hypokalemia, hypophosphatemia, hyponatremia, hypocalcemia and hypomagnesemia occurred frequently in patients with EHS. Renal function, serum CPK and neurological status at the time of admission should be considered as risk factors of acute dialysis. Therefore, rapid measurement of renal function, serum CPK and serum electrolytes might appropriately guide initial resuscitation in EHS patients.

## Abbreviations

AKI, Acute kidney injury; CI, Confidence interval; CPK, Creatine phosphokinase; EHS, Exertional heat stroke; FEMg, Fraction excretion of magnesium; FENa, Fraction excretion of sodium; FEPO_4_, Fraction excretion of phosphate; IL-6, Interleukin-6; urine K, Spot urine potassium
